# Access to pain treatment as a human right

**DOI:** 10.1186/1741-7015-8-8

**Published:** 2010-01-20

**Authors:** Diederik Lohman, Rebecca Schleifer, Joseph J Amon

**Affiliations:** 1 Health and Human Rights Division, Human Rights Watch, 350 Fifth Avenue, 34th Floor, NY 10118, USA

## Abstract

**Background:**

Almost five decades ago, governments around the world adopted the 1961 Single Convention on Narcotic Drugs which, in addition to addressing the control of illicit narcotics, obligated countries to work towards universal access to the narcotic drugs necessary to alleviate pain and suffering. Yet, despite the existence of inexpensive and effective pain relief medicines, tens of millions of people around the world continue to suffer from moderate to severe pain each year without treatment.

**Discussion:**

Significant barriers to effective pain treatment include: the failure of many governments to put in place functioning drug supply systems; the failure to enact policies on pain treatment and palliative care; poor training of healthcare workers; the existence of unnecessarily restrictive drug control regulations and practices; fear among healthcare workers of legal sanctions for legitimate medical practice; and the inflated cost of pain treatment. These barriers can be understood not only as a failure to provide essential medicines and relieve suffering but also as human rights abuses.

**Summary:**

According to international human rights law, countries have to provide pain treatment medications as part of their core obligations under the right to health; failure to take reasonable steps to ensure that people who suffer pain have access to adequate pain treatment may result in the violation of the obligation to protect against cruel, inhuman and degrading treatment.

## Background

Chronic pain is a one of the most significant causes of suffering and disability worldwide, and a common symptom of both cancer and HIV/AIDS. Up to 70% of cancer patients suffer from pain [1] and, among individuals living with HIV/AIDS, wide estimates of pain prevalence at all stages of infection have been reported [2-18]. While pain prevalence is diminished among individuals on antiretroviral therapy [16], studies continue to document the under-recognition and under-treatment of pain, even among individuals being treated for HIV infection [19-22]. Pain treatment is also related to gender, as HIV-infected women with pain are twice as likely to be under-treated as their male counterparts [21].

Pain has a profound impact on the quality of life and can have physical, psychological and social consequences. It can lead to reduced mobility and a consequent loss of strength, compromise the immune system and interfere with a person's ability to eat, concentrate, sleep, or interact with others [23]. A World Health Organization (WHO) study found that people who live with chronic pain are four times more likely to suffer from depression or anxiety [24,25]. The physical and psychological effects of chronic pain influence the course of disease [26]. Chronic pain can indirectly influence disease outcomes by reducing treatment adherence.

The WHO Pain Relief Ladder recommends the administration of different types of pain medications, depending on the severity of pain, and is the basis of modern pain management [27]. For mild pain, the WHO calls for basic pain relievers, usually widely available without prescription. For mild to moderate pain, it recommends a combination of basic pain relievers and a weak opioid, such as codeine. For moderate to severe pain, the WHO has recognized that strong opioids, such as morphine, are 'absolutely necessary' [28].

The WHO's recognition of the absolute necessity of opioid analgesics has reflected the consensus among health experts for decades. Almost 50 years ago, the United Nations (UN) member states adopted the 1961 *Single Convention on Narcotic Drugs*, declaring the medical use of narcotic drugs indispensable for the relief of pain and mandating an adequate provision of narcotic drugs for medical use [29]. The International Narcotics Control Board (INCB), charged with monitoring the implementation of the UN drug conventions, clarified in 1995 that the Convention 'establishes a dual drug control obligation: to ensure adequate availability of narcotic drugs, including opiates, for medical and scientific purposes, while at the same time preventing illicit production of, trafficking in and use of such drug' [30]. The WHO has included both morphine and codeine in its *Model List of Essential Medicines *[31]. Various other international bodies, such as the UN Economic and Social Council and the World Health Assembly, have also called on countries to ensure an adequate availability of opioid analgesics [32-35].

Yet, despite this clear consensus that pain treatment medications should be available, approximately 80% of the world population has either no, or insufficient, access to treatment for moderate to severe pain [36]. Millions of people living with cancer and HIV - including 1 million end-stage HIV/AIDS patients - suffer from moderate to severe pain each year without treatment [36].

Pain treatment medications are not evenly distributed worldwide. Approximately 89% of the total world consumption of morphine occurs in North America and Europe [37]. Low and middle income countries consume only 6% of the morphine used worldwide [38], even though they are home to about half of all cancer patients [26] and more than 90% of HIV infections [39]. However, inadequate pain management is also prevalent in developed countries. In the USA, the lack of the availability of pain medication in pharmacies, misinformation about addiction on the part of both patients and providers and fear of criminal sanctions for prescribing pain medicines are significant limiting factors [40]. Studies in Western Europe also document an under-estimation of pain severity and under-treatment of pain [17].

This article outlines the common obstacles to the provision of pain treatment and palliative care and calls for the reform of laws and policies inhibiting access to pain treatment worldwide. It also examines access to pain relief medicine in relation to the obligations of states under international human rights law. Identifying state obligations in the context of the HIV/AIDS epidemic has been a powerful mechanism for mobilizing attention and compelling response. By developing a human rights framework related to access to pain relief medicines, individuals living with HIV, as well as those with cancer or other causes of pain, can more effectively unite to demand the accountability of governments to respect, protect and fulfill their rights.

## Discussion

Barriers to access to pain treatment globally include: (1) the failure of governments to put in place functioning drug supply systems; (2) the failure to enact policies on pain treatment and palliative care; (3) poor training of healthcare workers; (4) the existence of unnecessarily restrictive drug control regulations and practices; (5) fear among healthcare workers of legal sanctions for legitimate medical practice; and (6) the unnecessarily high cost of pain treatment.

### Failure to ensure functioning and effective supply system

Opioid analgesics are controlled medicines and, as such, their manufacture, distribution and prescription are strictly regulated. The 1961 *Single Convention on Narcotic Drugs *has created a system for the regulation of supply and demand, overseen internationally by INCB and nationally by special drug control agencies. Every year, countries submit estimates of their need for morphine and other controlled medications to INCB, which approves each country's supply. Cross-border transactions must be authorized and registered by INCB.

As the production, distribution and dispensation of controlled medicines are under exclusive government control, governments must also put in place an effective system of distribution in order to provide healthcare providers and pharmacies with a continuous and adequate supply of the medications. Yet, many governments, as a result of resource limitations or lack of political will, have failed to put in place effective supply systems for controlled medicines [41]. More fundamentally, there is a lack of a common understanding of opiate pain relief needs and accessibility. A 2006 African Palliative Care Association survey found that, while drug control agencies in Kenya, Tanzania and Ethiopia believed the regulatory system worked well, morphine consumption in each of these countries was far below the estimated need and the palliative care providers surveyed identified myriad problems with the regulatory system [42].

While UN drug conventions require countries to submit estimates of their need for controlled substances based on a careful assessment of population needs, some countries either submit no estimates or submit only symbolic estimates. For example, the West African nation of Burkina Faso estimated that it would need 49 g of morphine in 2009 [43]. Based on an estimate for a terminal cancer or end-stage AIDS patient of a daily need for 70 mg of morphine for an average of 90 days, this amount would be sufficient for about eight patients. Even in countries that estimate a need for considerably greater absolute quantities of morphine, population needs are vastly underestimated (Table 1).

**Table 1:** Morphine estimates, mortality and pain treatment need.

Country	Cancer deaths 2002 estimate	AIDS deaths 2005 estimate	No. of individuals expected to need pain treatment in 2009	Estimated total morphine need in 2009 (kg)	Estimate of morphine need provided by country to INCB for 2009 (kg)	No. of individuals estimate is sufficient for	Percentage of those needing treatment who would be covered by estimate
**Countries that estimate almost no need for morphine**							
**Benin**	13490	9986	15786	96	0.5	83	0.50%

**Senegal**	17625	5432	16816	102	0.6	99	0.60%

**Rwanda**	14196	21956	22335	136	0.8	132	0.60%

**Gambia**	2395	1430	2631	16	0.18	31	1.20%

**Bhutan**	727	>10 per 100,000	582	3.5	0.08	14	2.30%

**Burkina Faso**	23262	13067	25143	153	0.05	8	0.03%

**Eritrea**	6240	5959	7972	48	0.075	12	0.15%

**Gabon**	2071	4457	3886	24	0.088	14	0.40%

**Swaziland**	1837	17577	10258	62	0.5	82	0.80%

**Selected other countries**							

**Egypt**	62299	>10 per 100,000	49840	303	10	1646	3%

**Philippines**	78500	>10 per 100,000	62800	382	31	5103	8%

**Kenya**	50809	149502	115398	701	30	4938	4%

**Russian Federation**	217696	N/A	174157	1058	200	32922	15%

**Mexico**	92701	6321	77321	470	180	29630	38%

Projection for the numbers of people requiring pain treatment does not include those with acute pain or pain related to non-terminal cancer or HIV and do not include pain control medications other than morphine. The table is based on an estimate by Foley and others that 80% of terminal cancer patients and 50% of terminal AIDS patients will require an average of 90 days of pain treatment with 60 mg to 75 mg of morphine per day [16]. Country estimates were obtained from INCB website [43]; projections for annual cancer and AIDS deaths are based on the most recent cancer and AIDS mortality figures reported by the WHO [79].

Without an effective distribution system, sufficient supply does not ensure accessibility. As controlled medications may only be transferred between parties that have been authorized under national law, governments must ensure that a sufficient number of pharmacies are licensed to handle morphine and that the procedures for procuring, stocking and dispensing it are practical.

Yet, in some cases, government regulations allow only a few institutions to stock the medication, sharply limiting accessibility [42]. In other countries, excessively burdensome procedures for procurement, dispensing and accounting effectively discourage health institutions from procuring the required morphine [44]. Where hospitals and pharmacies do stock morphine, interruptions in stock are common [42,45].

### Failure to enact palliative care and pain treatment policies

In 1996, the WHO identified the absence of national policies on cancer pain relief and palliative care as one of the reasons that cancer pain is not adequately treated [46]. In 2000, the organization noted that pain treatment continued to be a low priority in healthcare systems [28] and, again, in 2002 it noted that there was a wide gap between the rhetoric and the reality when integrating palliative care principles into public health and disease control programmes [26].

Although the WHO and leading experts on palliative care have stressed the importance of having a comprehensive strategy [47], most countries do not have palliative care and pain treatment policies, either as stand-alone policies or as part of cancer or HIV/AIDS control efforts [47,48]. Many countries have even failed to add oral morphine and other opioid-based medicines to their list of essential medicines or to issue guidelines on pain management for healthcare workers [42].

The INCB has recommended that national drug control laws recognize the indispensible nature of narcotic drugs for the relief of pain and suffering as well as the obligation to ensure their availability for medical purposes. However, in 1995 only 48% of the governments responding to a survey had laws reflecting the former and 63% the latter [30]. It is not known exactly how many countries still do not use the relevant language in their legislation and even recent model laws and regulations on drug control from the UN Office on Drugs and Crime themselves do not contain these provisions [49-52] (Appendix 1).

### Lack of training for healthcare workers

One of the biggest obstacles to the provision of good palliative and pain treatment services in many countries is the lack of training for healthcare workers. Misinformation about oral morphine is still extremely common among healthcare workers and knowledge of how to assess and treat pain is often very inadequate.

Some of the most common myths maintain that: treatment with opioids leads to addiction; that pain is necessary because it is enables diagnosis; that pain is unavoidable; and that pain has negligible consequences. Each of these myths is inaccurate. Numerous studies have shown that treatment of pain with opioids very rarely leads to addiction [28]; most pain can be treated well [28]; pain is not necessary for diagnosis [23]; and pain has considerable social, economic and psychological consequences as it prevents people who suffer from it, and often their caregivers, from living a productive life [23].

Throughout much of the world, including some industrialized countries, ignorance of the use of opioid medications is the result of a failure to provide healthcare workers with adequate training in palliative care and pain management. A survey by the Worldwide Palliative Care Alliance of healthcare workers in 69 countries in Latin America, Asia and Africa found that 82% in Latin America, 71% in Asia and 39% in Africa had not received any instruction on pain management or opioids during their undergraduate medical studies [53]. Additional studies have documented the significant number of healthcare providers in Africa who report inadequate opportunities for training in palliative care and pain treatment [42]. Even in industrialized countries instruction on palliative care and pain treatment remains a considerable challenge. A 1999 review found that considerable numbers of healthcare workers had insufficient factual knowledge about pain management among cancer patients in the industrialized countries [54] (Appendix 2).

### Excessively restrictive drug control regulations or enforcement practices

The 1961 the *Single Convention on Narcotic Drugs *lays out three minimum criteria that countries must observe when developing national regulations governing the handling of opioids. First, individuals must be authorized to dispense opioids by their professional license to practice or be specially licensed to do so. Secondly, movement of opioids may only occur between institutions or individuals so authorized under national law. Finally, a medical prescription is required before opioids may be dispensed to a patient. Governments may, under the Convention, impose additional requirements if deemed necessary [29].

However, many countries have regulations that go well beyond these restrictions, creating complex procedures for procurement, stocking and dispensing of controlled medications, including: restrictive licensing requirements for health care providers prescribing medicines; cumbersome dispensing procedures; and limitations on the formulation and quantity of medicine that can be prescribed [55]. In some cases, drug control authorities or health systems adopt even more restrictive measures than those required in the formal regulations. Although the diversion of medical opiods from its proper use is frequently cited as the explanation for such policies, the INCB has noted that, in practice, diversion is relatively rare [56] and the WHO has observed that 'this right [to impose additional requirements] must be continually balanced against the responsibility to ensure opioid availability for medical purposes' [46].

An example of overly restrictive policies adopted by many countries is to limit the prescription of narcotic pain medicines to medical professionals who qualify for specific licenses. The 1961 the *Single Convention on Narcotic Drugs *does not require healthcare workers to obtain a special license to handle opioids and the WHO has recommended that 'physicians, nurses and pharmacists should be legally empowered to prescribe, dispense and administer opioids to patients in accordance with local needs' [46]. Yet special licenses are common, and nurses and pharmacists are rarely able to prescribe pain medicines. For example, the Worldwide Palliative Care Alliance reported that, in 2007, in Mongolia, Peru, Honduras, Kyrgyzstan and a state in India only palliative care specialists and oncologists are authorized to prescribe oral morphine [53]. In Russia, an AIDS doctor reported that he could not treat a patient who suffered from severe pain because he was not licensed to prescribe morphine and that those oncologists who are would not provide treatment because the patient did not have cancer [57].

Another common obstacle is the special prescription procedures for opioids - for example the use of specific prescription forms and the insistence that multiple copies of the prescription be maintained. The WHO Expert Committee on Cancer Pain Relief has observed that these practices often reduce prescription of opioid pain medicines by 50% or more [46]. In 1995, INCB found that 65% of countries that participated in its survey had special prescription procedures [30].

Another special practice is the requirement that prescriptions by healthcare workers be approved by colleagues or superiors and that dispensing must be witnessed by multiple healthcare workers. In Ukraine, for example, the decision to prescribe morphine has to be made by a group of at least three doctors, one of whom must be an oncologist [58]. In South Africa, two nurses must observe the dispensing of opioids [42].

While the WHO has recommended that 'decisions concerning the type of drug to be used, the amount of the prescription and the duration of therapy are best made by medical professionals on the basis of the individual needs of each patient, not by regulation' [46], regulations imposing limitations on the dose of oral morphine that can be prescribed per day or the number of days for which it can be prescribed are common. The 1995 INCB survey found that 40% of countries participating set a maximum amount of morphine that could be prescribed at one time to a hospitalized patient and 50% of countries surveyed set limits for patients who lived at home [30]. Limits for home-based patients were frequently less than those for hospitalized patients - in some cases half of the typical daily doses [16]. The survey also found that 20% of participating countries imposed a maximum length of time that a hospitalized patient could receive morphine and 28% had such restrictions for patients at home. In some cases, prescriptions for morphine could not exceed a week's supply; in some countries this was non-renewable [30]. Although no recent comprehensive overview of countries that impose these kinds of limitations is available, they continue to be widespread [53,59].

### Fears of legal sanction

Although the INCB has recommended that healthcare workers be able to provide opiates without unnecessary fear of legal action for unintended violations which would inhibit provision or dispensation [60], ambiguity in regulations, poor communication by drug regulators to healthcare workers about the rules for handling opioids, the existence of harsh sanctions (including mandatory minimum sentences) and, in some countries, prosecutions of healthcare workers for unintentional mishandling of opioids, lead to fear among medical professionals. Little research has been published that documents the extent and impact of fears by healthcare providers on the prescription practice globally. A recent survey in the USA of criminal and administrative cases against physicians related to opiod prescription found an increasing trend in prosecutions which has a chilling effect on physician practice [61]. Some authors argue that physicians have an individual obligation to treat patients for severe pain and should be held accountable for a failure to treat pain via a charge of medical malpractice and, in extreme cases, criminal negligence [62].

### Cost

Although basic oral morphine is inexpensive [16], cost is a frequently cited impediment to improving access to pain treatment and palliative care services, particularly for low and middle income countries. A 2003 study found that the average retail cost of a monthly morphine supply ranged from US$10 in India to US$254 in Argentina [63]. Paradoxically, the study found that median cost of a month's supply of morphine was more than twice as high in low and middle income countries (US$112) as in industrialized countries (US$53). The study suggested that a number of factors might explain the discrepancy: medication subsidies by industrialized countries; industrialized government regulation of the price of opioids; taxes, licenses and other costs related to import of finished product; large overhead of local production; poorly developed distribution systems; low demand; and regulatory requirements that drive up cost. Further, a 2007 report also found that the promotion of non-generic - and costly - forms of opioid analgesics has made pain treatment medications unaffordable in some areas, as inexpensive formulations are withdrawn when more expensive opioids appear on the market [64].

A number of countries have successfully sought ways to create a capacity for the local production of basic oral morphine, in tablet or liquid form, at low cost. For example, in India, a small manufacturing unit has been set up at a hospital that produces low cost immediate release morphine tablets from morphine powder [64]. In Uganda, the ministry of health commissioned charitable procurement and manufacturing facility to produce morphine solution which could be distributed to hospitals, health centres and palliative care providers [65]. In Vietnam, a new opioid prescription regulation allows the ministry of health to mandate state and para-state pharmaceutical companies to produce oral and injectible opioids [66]. These examples illustrate the potential for creating locally manufactured, low-cost oral morphine.

### Health as a human right

Health is a fundamental human right enshrined in numerous international human rights instruments. The International Covenant on Economic Social and Cultural Rights (ICESCR) specifies that everyone has a right 'to the enjoyment of the highest attainable standard of physical and mental health' [67]. The Committee on Economic, Social and Cultural Rights, the treaty's monitoring body, has held that states must make available and accessible in sufficient quantity 'functioning public health and health-care facilities, goods and services, as well as programmes' [68].

As states have different levels of resources, international law does not mandate the kind of health care to be provided and, instead, demands 'progressive realization'. By committing to the international agreements, a state agrees 'to take steps... to the maximum of its available resources' to achieve the full realization of the right to health. High income countries will generally have to provide healthcare services at a higher level than those with limited resources, but all countries will be expected to take concrete steps toward increased services.

The Committee on Economic, Social and Cultural Rights has also held that there are certain core obligations that are so fundamental that states must fulfill them. While resource constraints may justify only partial fulfillment of some aspects of the right to health, the Committee has observed that 'a State party cannot, under any circumstances whatsoever, justify its non-compliance with the core obligations...which are nonderogable'. The Committee has identified, among others, the following core obligations: to ensure the right of access to health facilities, goods and services on a non-discriminatory basis, especially for vulnerable or marginalized groups; to provide essential drugs, as from time to time defined under the WHO Action Programme on Essential Drugs; to ensure the equitable distribution of all health facilities, goods and services; and to adopt and implement a national public health strategy and plan of action, on the basis of epidemiological evidence, addressing the health concerns of the whole population [68].

### Pain treatment and the right to health

As morphine and codeine are on the WHO List of Essential Medicines [31], countries have to provide these medications as part of their core obligations under the right to health, regardless of whether or not they have been included on their domestic essential medicines lists [68]. Countries must ensure that they are both available in adequate quantities and physically and financially accessible for those who need them.

Since manufacturing and distribution of controlled medicines, such as morphine and codeine, are completely in government hands, states must put in place an effective procurement and distribution system and create a legal and regulatory framework that enables healthcare providers in both the public and private sectors to obtain, prescribe and dispense these medications. Any regulations that arbitrarily impede the procurement and dispensing of these medications will violate the right to health. States must adopt and implement a strategy and plan of action for the roll out of pain treatment and palliative care services. Such a strategy and plan of action should identify obstacles to improved services as well as take steps to eliminate them.

States should also regularly measure progress made in ensuring the availability and accessibility of pain relief medications. The requirement of physical accessibility means that these medications must be 'within safe physical reach for all sections of the population, especially vulnerable or marginalized groups, such as...persons with HIV/AIDS' [68]. This means that states must ensure that a sufficient number of healthcare providers or pharmacies stock and dispense morphine and codeine and that an adequate number of healthcare workers are trained and authorized to prescribe these medications. Financial accessibility means that, while the right to health does not require states to offer medications free of charge, they must be 'affordable for all'. According to the Committee, payment for healthcare services must be based on equity and poorer households should not be disproportionately burdened by cost [68].

Countries also have an obligation to progressively implement palliative care services, which, according to the WHO, must have 'priority status within public health and disease control programmes' [26]. Countries must ensure an adequate policy and regulatory framework, develop a plan for the implementation of these services and take all steps that are reasonable within available resources to execute the plan. Failure to attach adequate priority to developing palliative care services within healthcare services will violate the right to health [55].

### Pain treatment and the right to be free from cruel, inhuman and degrading treatment

The right to be free from torture, cruel, inhuman and degrading treatment or punishment is also a fundamental human right that is recognized in numerous international human rights instruments [69-75]. This right creates a positive obligation for states to protect persons in their jurisdiction from torture, cruel, inhuman and degrading treatment or punishment. In a letter to the Chairperson of the 52nd Session of the Commission on Narcotic Drugs, the UN Special Rapporteur on Torture, Cruel, Inhuman and Degrading Treatment and Punishment and the UN Special Rapporteur on the Right to Health noted that governments' failure to take measures to ensure the accessibility of pain treatment threatens this fundamental right [75].

### Summary

The lack of pain treatment medicine is both perplexing and inexcusable. Pain causes terrible suffering yet the medications to treat it are cheap, safe, effective and generally straightforward to administer. Furthermore, international law obliges countries to make adequate pain medications available. Over the last 20 years, the WHO and the INCB have repeatedly reminded states of this obligation. However, little progress has been made and tens of millions of people continue to suffer - both directly from untreated pain and from its consequences.

Under international human rights law, governments must take steps to ensure that people have adequate access to treatment for their pain. At a minimum, states must ensure the availability of morphine, the mainstay medication for the treatment of moderate to severe pain. Failure to make essential medicines such as morphine available or, more broadly, to take reasonable steps to make pain management and palliative care services accessible to all, results in a violation of the right to health. In some cases, failure to ensure patients have access to treatment for severe pain will also give rise to a violation of the prohibition of cruel, inhuman and degrading treatment.

There are many reasons for the enormity of the gap between pain treatment needs and what is delivered, but the chief among them is a willingness by many governments around the world to passively stand by as people suffer. Excessive over-regulation by governments and the ignorance of healthcare providers conspire to create a vicious cycle of under-treatment. As pain treatment and palliative care are not priorities for the government, healthcare workers do not receive the necessary training in order to assess and treat pain. This leads to widespread under-treatment and to a low demand for morphine. Similarly, complex procurement and prescription regulations, and the threat of harsh punishment for mishandling morphine, discourage pharmacies and hospitals from stocking and healthcare workers from prescribing it, which again results in low demand. A lack of the prioritization of opioid pain medicine is not a result of the low prevalence of pain but of the invisibility of its sufferers.

To break out of this vicious cycle, individual governments and the international community must fulfill their obligations under international human rights law. Governments must take action to eliminate barriers that impede the availability of pain treatment medications. They must develop policies on pain management and palliative care; introduce instruction for healthcare workers, including for those already practicing; reform regulations that unnecessarily impede the accessibility of pain medications; and take action to ensure their affordability. While this is a considerable task, various countries, such as Uganda and Vietnam, have shown that such a comprehensive approach is feasible in low and middle-income countries. Other nations must learn from these experiences and work towards the realization of full access to pain relief medicines.

## Competing interests

The authors declare they have no competing interests. This research was supported by Human Rights Watch, an independent, nongovernmental organization.

## Authors' contributions

DL and JJA conceived the article and reviewed the existing literature. DL wrote the article and RS and JJA reviewed and contributed to a revised version. All authors reviewed and approved the final text.

## Appendix 1: Uganda case study

In 1998, Ugandan government officials, representatives of non-governmental organizations and the WHO agreed on ways in which pain treatment could be made available to the population. These steps included: developing national palliative care, cancer and AIDS pain relief policies; implementing a training course to complement existing palliative medicine teaching and increasing the number of skilled providers; developing new drug regulations: updating the essential drug list; conducting estimates of the medical need for morphine; and requests from the drug control authority for an increased national allowance from the INCB [77].

Following this agreement, Uganda has made considerable progress in reducing or eliminating barriers that have traditionally impeded access to pain treatment medications. In its 5-year Strategic Health Plan for 2000-2005, the government noted that palliative care was an essential clinical service for all Ugandans and so became the first nation in Africa to do so. It also added liquid morphine to its essential drug list, adopted a new set of guidelines for the handling of class A drugs for healthcare practitioners - also a first in Africa - and authorized the prescribing of morphine by nurses who have been trained in palliative care. By early 2009, 79 nurses and clinical officers had received training in pain management and been authorized to prescribe oral morphine, several thousand healthcare workers had attended a short course on pain and symptom management and 34 out of 56 districts in Uganda had oral morphine available and in use [65]. Despite this impressive progress, many challenges remain, including: ensuring the availability of oral morphine throughout Uganda; keeping it affordable; preventing stockouts; and training all relevant healthcare workers.

## Appendix 2: Vietnam case study

Since 2005, Vietnam has made considerable progress in expanding access to palliative and pain treatment services. A working group on palliative care, including health officials, physicians and NGOs, conducted a rapid situation analysis devised to assess the availability of, and need for, palliative care in Vietnam. This rapid analysis found severe chronic pain to be common among cancer and HIV/AIDS patients, while the availability of opioid analgesics was severely limited, palliative care services were not readily available and clinicians lacked adequate training [78]. The working group recommended that national palliative care guidelines be developed, a balanced national opioid control policy be designed, training for healthcare workers be expanded and that the availability and quality of palliative care services be improved.

In 2006, the Ministry of Health issued detailed guidelines to practitioners on palliative care and pain management and, in 2008, it issued new guidelines on opioid prescription which have eased a number of key regulatory barriers. The Ministry has also approved a package of training courses for practicing physicians and two medical colleges now offer instruction on palliative care to undergraduate medical and nursing students. However, only a few hundred healthcare workers have received training so far, understanding of palliative care among healthcare officials continues to be limited, various regulatory barriers persist and few pharmacies and hospitals stock oral morphine.

## Pre-publication history

The pre-publication history for this paper can be accessed here:

http://www.biomedcentral.com/1741-7015/8/8/prepub

## References

[B1] Beuken-van Everdingen MHJ Van den De Rijke JM Kessels AG Schouten HC Van Kleef M Patjin J Prevalence of pain in patients with cancer: a systematic review of the past 40 years Ann Onc 2007 18 1437 1449 10.1093/annonc/mdm056 17355955

[B2] McCormack JP Li R Zarowny D Singer J Inadequate treatment of pain in ambulatory HIV patients Clin J Pain 1993 9 279 283 8118093 10.1097/00002508-199312000-00010

[B3] Singer EJ Zorilla C Fahy-Chandon B Chi S Syndulko K Tourtelotte WW Painful symptoms reported by ambulatory HIV infected men in a longitudinal study Pain 1993 54 15 19 10.1016/0304-3959(93)90094-6 8378098

[B4] Lebovits AH Smith G Maignan M Lefkowitz M Pain in hospitalized patients with AIDS: analgesic and psychotropic medications Clin J Pain 1994 10 156 161 8075469 10.1097/00002508-199406000-00010

[B5] Shofferman J Pain: diagnosis and management in the palliative care of AIDS J Palliat Care 1988 4 46 49 2463359

[B6] Moss V Patient characteristics, presentation and problems encountered in advanced AIDS in a hospice setting: a review Palliat Med 1991 5 112 116

[B7] Dixon P Higinson A AIDS and cancer pain treated with slow release morphine Postgrad Med J 1991 67 S92 S94 10.1136/pgmj.67.788.557 1758826

[B8] Martin C Pehrsson P Osterberg A Sonnerborg A Hansson P Pain in ambulatory HIV-infected patients with and without intravenous drug use Eur J Pain 1999 3 157 164 10.1053/eujp.1999.0111 10700345

[B9] Breitbart W Rosenfeld BD Passik SD McDonald MV Thaler H Portenoy RK The undertreatment of pain in ambulatory AIDS patients Pain 1996 65 243 249 10.1016/0304-3959(95)00217-0 8826513

[B10] Fantoni M Ricci F Del Borgo C Izzi I Damiano F Moscati AM Marasca G Bevilacqua N Del Forno A Multicentre study on the prevalence of symptoms and symptomatic treatment in HIV infection J Palliat Care 1997 13 9 13 9231582

[B11] Fontaine A LaRue F Lasauniere JM Physicians' recognition of the symptoms experienced by HIV patients: how reliable? J Pain Symptom Manage 1999 18 263 270 10.1016/S0885-3924(99)00078-0 10534966

[B12] Mathews W McCutcheon JA Asch S Turner BJ Gifford AL Kuromiya K Brown J Shapiro MF Bozzette SA National estimates of HIV-related symptom prevalence from the HIV Cost and Services Utilization Study Med Care 2000 38 762 10.1097/00005650-200007000-00007 10901358

[B13] Vogl D Rosenfeld B Brietbart W Thaler H Passik S McDonald M Portenoy RK Symptom prevalence, characteristics, and distress in AIDS outpatients J Pain Symptom Manage 1998 18 253 262 10.1016/S0885-3924(99)00066-4 10534965

[B14] Selwyn PA Rivard M Kappell D Goeren B LaFosse H Schwartz C Caraballo R Luciano D Post LF Palliative care for AIDS at a large urban teaching hospital: program description and preliminary outcomes J Palliat Med 2003 6 461 474 10.1089/109662103322144844 14509496

[B15] Harding R Molloy T Easterbrook PE Frame K Higginson IJ Is antiretroviral therapy associated with symptom prevalence and burden? Int J STD AIDS 2006 17 400 405 10.1258/095646206777323409 16734963

[B16] Foley KM Wagner JL Joranson DE Gelband H Pain control for people with cancer and AIDS Disease Control Priorities in Developing Countries 2003 2 New York: Oxford University Press 981 994

[B17] Larue F Fontaine A Colleau SM Underestimation and under-treatment of pain in HIV disease: a multicentre study BMJ 1997 314 23 9001475 10.1136/bmj.314.7073.23 PMC2125594

[B18] Dalakas MC Peripheral neuropathy and antiretroviral drugs J Periph 2001 6 14 20 10.1046/j.1529-8027.2001.006001014.x 11293802

[B19] Breitbart W Patt RB Passik SD Reddy KS Lefkowitz M Pain: clinical updates IASP 2006 4 1 8

[B20] Harding R Easterbrook P Dinat N Higginson IJ Pain and symptom control in HIV disease: under-researched and poorly managed Clin Infect Dis 2005 40 491 492 10.1086/427037 15668879

[B21] Hewitt D McDonald M Portenoy R Rosenfeld B Passik S Breitbart W Pain syndromes and etiologies in ambulatory AIDS patients Pain 1997 70 117 123 10.1016/S0304-3959(96)03281-2 9150284

[B22] Patel P Hanson DL Sullivan PS Nowak RM Moorman AC Tong TC Holmberg SD Brooks JT Adult and Adolescent Spectrum of Disease Project and HIV Outpatient Study Investigators Incidence of types of cancer among HIV-infected persons compared with the general population in the United States, 1992-2003 Ann Intern Med 2008 148 728 736 18490686 10.7326/0003-4819-148-10-200805200-00005

[B23] Brennan F Carr DB Cousins MJ Pain management: a fundamental human rights Anesth Analg 2007 105 205 221 10.1213/01.ane.0000268145.52345.55 17578977

[B24] Gureje O Von Korff M Simon GE Gater R Persistent pain and well-being: a World Health Organization study in primary care JAMA 1998 80 147 151 10.1001/jama.280.2.147 9669787

[B25] Rosenfeld B Breitbart W McDonald MV Passik SD Thaler H Portenoy RK Pain in ambulatory AIDS patients. II: Impact of pain on psychological functioning and quality of life Pain 1996 68 323 328 10.1016/S0304-3959(96)03220-4 9121821

[B26] World Health Organization National Cancer Control Programme: Policies and Managerial Guidelines 2002 Geneva: WHO

[B27] O'Neill JF Selwyn PA Schietinger H A Clinical Guide to Supportive and Palliative Care for HIV/AIDS 2003 Washington, DC: Health Resources and Services Administration

[B28] World Health Organization Achieving Balance n National Opioids Control Policy 2000 Geneva: WHO

[B29] United Nations Single Convention on Narcotic Drugs 1961 UN http://www.incb.org/incb/convention_1961.html

[B30] International Narcotics Control Board Availability of Opiates for Medical Needs: Report of the International Narcotics Control Board for 1995 1996 INCB http://www.incb.org/pdf/e/ar/1995/suppl1en.pdf

[B31] World Health Organization WHO Model List of Essential Medicines 2007 15 Geneva: WHO http://www.who.int/medicines/publications/08_ENGLISH_indexFINAL_EML15.pdf

[B32] United Nations Economic and Social Council Resolution 2005/25: Treatment of Pain Using Opioid Analgesics 2005 UN http://www.un.org/docs/ecosoc/documents/2005/resolutions/Resolution%202005-25.pdf

[B33] United Nations Economic and Social Council Resolution 1990/31 1990

[B34] United Nations Economic and Social Council Resolution 1991/43 1991

[B35] World Health Assembly Resolution WHA 58.22 on Cancer Prevention and Control 2005 UN http://www.who.int/gb/ebwha/pdf_files/WHA58/WHA58_22-en.pdf

[B36] World Health Organization Briefing Note: Access To Controlled Medications Programme 2008 Geneva: WHO

[B37] International Narcotics Control Board Report of the International Narcotic Control Board for 2007. E/INCB/2007/1 2008 INCB

[B38] International Narcotics Control Board Report of the International Narcotics Control Board for 2004 2005 INCB

[B39] UNAIDS Report on The Global AIDS Epidemic 2008 2008 UNAIDS

[B40] Foley KM Ideas For an Open Society: Pain Management 2002

[B41] International Narcotics Control Board Report of the International Narcotic Control Board for 1999. E/INCB/1999/1 1999 INCB

[B42] Harding R Powell RA Kiyange F Downing J Mwangi-Powell F Pain-Relieving Drugs in 12 African PEPFAR Countries: Mapping Current Providers, Identifying Current Challenges and Enabling Expansion of Pain Control Provision in the Management of HIV/AIDS 2007 Kampala: African Palliative Care Association

[B43] International Narcotics Control Board Narcotic Drugs Estimated World Requirements of Narcotic Drugs for 2009. E/INCB/2008/2 2009 INCB

[B44] Email correspondence with Liliana de Lima, Executive Director of the International Hospice and Palliative Care Association 2009

[B45] Interview with Liliana de Lima, Executive Director of the International Hospice and Palliative Care Association 2009

[B46] World Health Organization Cancer Pain Relief: a Guide To Opioid Availability 1996 2 Geneva: WHO

[B47] Stjernsward J Clark D Doyle G, Hanks WC, Cherny N, Calman K Palliative medicine: a global perspective Oxford Textbook of Palliative Medicine 2003 New York: Oxford University Press 1199 222

[B48] DFID Health Resource Center Review of Global Policy Architecture and Country Level Practice on HIV/AIDS and Palliative Care 2007 DFID

[B49] United Nations Office on Drugs and Crime Model Drug Laws Designed For Civil Law Countries: Document A - Model Law On The Classification of Narcotic Drugs, Psychotropic Substances and Precursors and on the Regulation of the Licit Cultivation, Production, Manufacture and Trading of Drugs 2003 UN

[B50] United Nations Office on Drugs and Crime Model Regulation Establishing an Interministerial Commission for the Coordination of Drug Control 2002 UN

[B51] Model Drug Abuse Bill http://www.unodc.org/unodc/en/legal-tools/Model.html

[B52] Do international model drug control laws provide for drug availability? http://www.painpolicy.wisc.edu/internat/model_law_eval.pdf 10.1080/15360280902900729 19492215

[B53] Adams V Access to pain relief - an essential human right J Pain Palliat Care Pharmacother 2008 22 101 129 10.1080/15360280801992025 19062351

[B54] Green K Kinh LN Khue LN Palliative Care in Vietnam: Findings from a Rapid Situation Analysis in Five Provinces 2006 Hanoi: Vietnam Ministry of Health

[B55] Human Rights Watch Unbearable Pain: India's Obligation to Ensure Palliative Care 2009 HRW http://www.hrw.org/en/node/86153

[B56] International Narcotics Control Board Report of the International Narcotic Control Board for 2008 2009 United Nations

[B57] Proceedings of the 2nd Eastern Europe and Central Asia AIDS Conference: 3-5 May 2008. Moscow 2008

[B58] The Ministry of Health of Ukraine Order No. 356

[B59] Anderson E Beletsky L Burris S Davis C Kresina T eds Closing the gap: case studies of opioid access reform in China, India, Romania and Vietnam 2008 Report for the UK Department of International Development. DID

[B60] International Narcotics Control Board Demand For and Supply of Opiates for Medical and Scientific Needs 1989 INCB

[B61] Goldenbaum DM Christopher M Gallagher RM Fishman S Payne R Joranson D Edmondson D McKee J Thexton A Physicians charged with opioid analgesic-prescribing offenses Pain Medicine 2008 9 737 47 10.1111/j.1526-4637.2008.00482.x 18657218

[B62] Margaret Somerville Gerald F Gebhart, Donna L Hammond, Troels S Jensen Death of pain: pain, suffering and ethics Progress in Pain Research and Management, Proceedings of the 7th World Congress on Pain, International Association for the Study of Pain 1994 2 Washington: IASP Press 41 58

[B63] De Lima L Sweeney C Palmer JL Bruera E Potent analgesics are more expensive for patients in developing countries: a comparative study J Pain Palliat Care Pharmacother 2004 18 63 15148009

[B64] Joransen DE Rajagopal MR Gilson AM Improving access to opioid analgesics for palliative care in India J Pain Symptom Manage 2002 24 152 59 10.1016/S0885-3924(02)00458-X 12231133

[B65] Email correspondence with Anne Merriman, Hospice Uganda 2009

[B66] Email correspondence with Kimberly Green, Family Health International Vietnam 2009

[B67] International Covenant on Economic, Social and Cultural Rights GA Res. 2200A (XXI). 21 U.N. GAOR Supp. No. 16 at 49. U.N. Doc. A/6316. 993 U.N.T.S. 3. art. 11 1966

[B68] UN Committee on Economic, Social and Cultural Rights 2000 General Comment No. 14: The Right To The Highest Attainable Standard Of Health. UN

[B69] International Covenant on Civil and Political Rights GA Res. 2200A (XXI). 21 UN GAOR Supp. No. 16 at 52. UN Doc. A/6316. art. 7. UN 1966

[B70] UN Universal Declaration of Human Rights UN Doc A/810. UN 1948

[B71] Convention Against Torture and Other Cruel, Inhuman or Degrading Treatment or Punishment GA Res. 39/46, Annex. 39 UN GAOR Supp. No. 51 at 197. UN Doc. A/39/51. art. 16. UN 1984

[B72] Inter-American convention to prevent and punish torture OAS Treaty Series No. 67. OAS 1987

[B73] European Convention for the Prevention of Torture and Inhuman or Degrading Treatment or Punishment ETS 126 1987

[B74] African [Banjul] Charter on Human and Peoples' Rights OAU Doc. CAB/LEG/67/3 rev. 5. 21 I.L.M. 58 1981

[B75] Z v. United Kingdom. 4 EHRR 97 2001

[B76] Letter from Special Rapporteur on the question of torture and Special Rapporteur on the right of everyone to the enjoyment of the highest attainable standard of physical and mental health to Ms. Selma Ashipala-Musavyi, Chairperson of the 52nd Session of the Commission on Narcotic Drugs 2008

[B77] Stjernsward J Uganda: initiating a government public health approach to pain relief and palliative care J Pain Symptom Manage 2002 24 10.1016/S0885-3924(02)00451-7 12231159

[B78] Green K Kinh LN Khue LN Palliative care in Vietnam: Findings from a rapid situation assessment in five provinces 2006 Hanoi: Vietnam Ministry of Health

[B79] WHO Statistical Information System http://www.who.int/whosis/en/

